# Adherence to the Atrial Fibrillation Better Care (ABC) pathway and the risk of major outcomes in patients with atrial fibrillation: A post-hoc analysis from the prospective GLORIA-AF Registry

**DOI:** 10.1016/j.eclinm.2022.101757

**Published:** 2022-11-24

**Authors:** Giulio Francesco Romiti, Marco Proietti, Niccolò Bonini, Wern Yew Ding, Giuseppe Boriani, Menno V. Huisman, Gregory Y.H. Lip, GLORIA-AF Investigators

**Affiliations:** a Liverpool Centre for Cardiovascular Science, at University of Liverpool, Liverpool John Moores University and Liverpool Heart & Chest Hospital, Liverpool, United Kingdom; b Department of Translational and Precision Medicine, Sapienza – University of Rome, Rome, Italy; c Department of Clinical Sciences and Community Health, University of Milan, Milan, Italy; d Geriatric Unit, IRCCS Istituti Clinici Scientifici Maugeri, Milan, Italy; e Cardiology Division, Department of Biomedical, Metabolic and Neural Sciences, University of Modena and Reggio Emilia, Policlinico di Modena, Modena, Italy; f Department of Thrombosis and Hemostasis, Leiden University Medical Center, Leiden, the Netherlands; g Department of Clinical Medicine, Aalborg University, Aalborg, Denmark

**Keywords:** Atrial fibrillation, Integrated care, Outcomes

## Abstract

**Background:**

The ‘Atrial fibrillation Better Care’ (ABC) pathway has been proposed to streamline a more holistic or integrated care approach to atrial fibrillation (AF) management. We aimed to analyse the impact of adherence to the ABC pathway on the risk of major adverse outcomes in a contemporary prospective global cohort of patients with AF.

**Methods:**

Patients enrolled Phase II and III of the GLORIA-AF Registry with complete data on ABC pathway adherence and follow-up were included in this post-hoc analysis between November 2011 and December 2014 for Phase II, and between January 2014 and December 2016 for Phase III. The primary outcome was the composite of all-cause death and major adverse cardiovascular events (MACEs). Multivariable Cox-regression and delay of event (DoE) analyses were used to evaluate the association between adherence to the ABC pathway and the risk of outcomes.

**Findings:**

We included 24,608 patients in this analysis (mean age: 70.2 (10.3) years, 10,938 (44.4%) females). Adherence to the ABC pathway was associated with a significant risk reduction for the primary outcome, with greatest magnitude observed for full ABC pathway adherence (adjusted Hazard Ratio [aHR] 0.54, 95% Confidence Interval [CI]: 0.44–0.67, p < 0.0001). ABC pathway adherence was also associated with reduced risk of mortality (aHR: 0.89, 95% CI: 0.79–1.00, p = 0.048), thromboembolism (aHR: 0.78, 95% CI: 0.65–0.94, p = 0.0078), and MACE (aHR: 0.82, 95% CI: 0.71–0.95, p = 0.0071). An increasing number of ABC criteria attained was associated with longer event-free survival in the DoE analysis.

**Interpretation:**

Adherence to the ABC pathway in patients with AF was associated with a reduced risk of major adverse events, including mortality, thromboembolism and MACE. This underlines the importance of using the ABC pathway in the clinical care of patients with AF.

**Funding:**

This study was funded by Boehringer Ingelheim.

**Research in context**

**Evidence before this study**

The ‘Atrial fibrillation Better Care’ (ABC) pathway has been proposed to streamline the implementation of an integrated and holistic approach to atrial fibrillation (AF) care. We searched PubMed from inception to September 15, 2022, without language restrictions, for randomised trials, systematic reviews, meta-analyses, and observational studies, using the terms “Atrial Fibrillation” and “ABC pathway” or “integrated care”. The effectiveness of the ABC pathway on improving outcomes in patients with AF has been previously shown, although focused on specific regions and on cardiovascular comorbidity optimisation.

**Added value of this study**

In this large, global contemporary cohort of around 24,000 patients with AF, we found that adherence to the ABC pathway was associated with a significant reduction in the risk of major adverse outcome; the effect was higher as the number of ABC criteria attained increased. Similar results were observed when other non-cardiovascular comorbidities were considered in the ABC pathway.

**Implications of all the available evidence**

Our findings suggested that adherence to the ABC pathway is associated with improved prognosis in patients with AF, with reduced mortality, thromboembolism and major adverse cardiovascular events. Ensuring implementation of the ABC pathway is pivotal to provide better care and improve the prognosis of patients with AF.

## Introduction

Atrial Fibrillation (AF) represents the worldwide most common arrhythmia, with prevalence increasing globally and projected to reach over 17 million individuals in Europe and 72 million in Asia by 2050.^1^, ^2^, ^3^ Improvements in the management of AF have reduced the risks of thromboembolic and cardiovascular events, but mortality, hospitalisations and healthcare costs are still high in patients with AF.^4^, ^5^, ^6^

One of the main barriers to improving prognosis in patients with AF is their increasing clinical complexity, as encompassed by progressive aging, multimorbidity and polypharmacy,^7^^,^^8^ which contribute to worse prognosis. In this light, the need for a more holistic or comprehensive approach to the management of patients with AF has been advocated as a key step to improve their care and prognosis.

The ‘Atrial fibrillation Better Care’ (ABC) pathway has been proposed as an approach to streamline integrated care in patients with AF,^9^ and is based on three pillars: ‘A’, anticoagulation/avoid stroke; ‘B’, better symptom control, and ‘C’, cardiovascular risk factors and comorbidities optimization. Retrospective studies have shown that the ABC pathway is effective in reducing the risk of adverse outcomes among patients with AF.^10^, ^11^, ^12^ These beneficial findings on clinical outcomes were prospectively tested in the mAFA-II cluster randomised trial^13^ and further supported by a recent systematic review and meta-analysis^14^ and cost-effectiveness analysis.^15^ Given such strong evidence, recent international AF guidelines^16^^,^^17^ have recommended the implementation of such an integrated care approach for the management of AF, based on the ABC pathway.

Previous analyses on the impact of the ABC pathway were confined to specific countries or regions,^14^ and have focused mainly on *cardiovascular* comorbidity optimization (rather than non-cardiovascular comorbidities) for the ‘C’ criterion of the ABC pathway and the effect of its overall adherence.

In this analysis, using data from a contemporary prospective global cohort of patients with AF, the Global Registry on Long-Term Oral Anti-thrombotic Treatment in Patients with Atrial Fibrillation (GLORIA-AF) registry Phase II and III, our aims were as follows: i) to evaluate the impact of adherence to different number of ABC pathway criteria on the risk of outcomes; and ii) to explore whether the expansion of adherence to the non-cardiovascular comorbidities pool for the ‘C’ criterion would provide consistent estimates on the impact of the ABC pathway in patients with AF.

## Methods

### Study design

Full details on the design and protocols of the GLORIA-AF study have been previously reported.^18^, ^19^, ^20^ Briefly, GLORIA-AF is an international, multicentre prospective registry program composed of 3 phases, aimed at evaluating the long-term safety and effectiveness of dabigatran etexilate in real-world patients with AF. Consecutive patients with new-onset non-valvular AF and CHA_2_DS_2_-VASc score ≥1 were recruited between November 2011 and December 2014 for Phase II, and between January 2014 and December 2016 for Phase III. Patients who received dabigatran during Phase II were followed-up for the onset of major outcomes over 2 years, while all participants enrolled during the Phase III of the program were followed-up, irrespective of the antithrombotic treatment received, for 3 years. The primary papers from GLORIA-AF Phase III registry comparing the different anticoagulation strategies have recently been published.^21^^,^^22^

### Inclusion/exclusion criteria and procedures

Eligible patients for the inclusion in the GLORIA-AF registry were patients with AF aged 18 years or older, with a recent diagnosis of AF (<3 months, except in Latin America where <4.5 months cut-off was used) and a CHA_2_DS_2_-VASc score ≥1, who provided written informed consent. Detailed inclusion and exclusion criteria were described elsewhere^22^; briefly, main exclusion criteria were: mechanical heart valve or expected valve replacement; having received more than 60 days of VKA treatment in the lifetime; having other medical indication for oral anticoagulant (OAC) treatment; life expectancy <1 year; or AF due to a reversible cause. The protocol of the study was approved by the European Medicines Agency, and the study was conducted in accordance with the principles of Good Clinical Practice and the Declaration of Helsinki. Local institutional review boards at each participating site gave ethical approval. For this analysis, we included those patients with complete data to evaluate retrospectively the adherence to the ABC pathway, and follow-up data on the primary outcome defined for this analysis, which was the composite outcome of all-cause death and major adverse cardiovascular events (MACEs).

### Assessment of adherence to the ABC pathway

We defined two versions of the ABC pathway, which differed according to the ‘C’ criterion definition. For our *primary analysis*, we defined a ‘standard’ version of the ABC pathway, in which we evaluated adherence to ‘C’ according to the most common comorbidities found in patients with AF^23^^,^^24^; for the exploratory secondary analysis on the ‘expanded’ version of the ABC pathway, we also evaluated 3 additional *non-cardiovascular comorbidities* (hyperthyroidism, hyperlipidaemia, dyspeptic disease).

Adherence to each criterion of the ABC pathway was defined as follows:

- ‘A’ Criterion: patients were considered adherent to this criterion if appropriately prescribed at baseline with OAC according to the baseline thromboembolic risk. Male patients with CHA_2_DS_2_-VASc ≥1 and females with CHA_2_DS_2_-VASc ≥2 who received an OAC (either vitamin K antagonist [VKA] or a non-vitamin K antagonist oral anticoagulant [NOAC]) were considered adherent to this criterion, as well as those with low thromboembolic risk (i.e., CHA_2_DS_2_-VASc 0 in males or 1 in females) who did not receive OAC.
- ‘B’ Criterion: according to the original definition of the ABC pathway, actual symptoms control was evaluated through the EHRA score at baseline. Patients with EHRA score I (no symptoms) or II (mild symptoms) were considered adherent to this criterion.
- ‘C’ criterion: Assessment was made according to the presence and treatment of baseline comorbidities. For the ‘standard’ version of the ‘C’ criterion, we considered the comorbidities which are most commonly found in patients with AF and that were previously used to evaluate adherence to the ABC pathway^12^: hypertension, diabetes, coronary artery disease (CAD), congestive heart failure (CHF), history of previous stroke/TIA and peripheral artery disease (PAD). For the exploratory “expanded” version, we additionally evaluated the presence and treatment of dyslipidaemia, dyspeptic disease (as defined by the presence of gastritis/duodenitis or peptic ulcer) and hyperthyroidism.

Details on the optimal treatment definitions are reported in Supplementary Table S1. Adherence to the ‘C’ criterion was calculated only for those patients with data on at least 5 out of 6 comorbidities for the ‘standard’ version, and 7 out of 9 comorbidities for the ‘expanded’ version; patients without comorbidities, and those with optimal treatment of all comorbidities, were considered adherent to this criterion, while the others were considered non-adherent.

In the primary analysis, patients were divided according to the number of ABC criteria fulfilled, from 0 (none) to 3 (all criteria). For our secondary analyses, we also considered a) adherence to 0–1 vs. 2–3 ABC criteria; b) full-adherence vs. non-adherence to ABC pathway; and c) pattern of ABC criteria adherence.

### Major adverse outcomes

Details on follow-up and outcomes for GLORIA-AF Phase II and Phase III were reported elsewhere.^22^^,^^25^ For this analysis, we defined our primary outcome as the composite of all-cause death and MACEs (defined as the occurrence of cardiovascular death, stroke, and myocardial infarction). We also investigated the impact of ABC pathway on the risk of the following exploratory outcomes:

- stroke (including hemorrhagic, ischemic, and uncertain classification strokes);
- thromboembolism (as the composite of stroke, transient ischemic attack, and other non-central nervous system thromboembolism),
- major bleeding (defined according to the International Society of Thrombosis and Haemostasis classification, i.e. overt bleeding associated with a hemoglobin reduction of at least 20 g/L or leading to at least 2-unit of blood transfusion, symptomatic bleeding in a critical organ, life-threatening or fatal bleeding);
- myocardial infarction (MI);
- MACEs,
- All-cause death;
- cardiovascular death;
- The composite of all-cause death, stroke and major bleeding;
- The composite of all-cause death, MACE and major bleeding.

### Statistical analysis

Baseline characteristics were reported as mean and standard deviation (SD) or median and interquartile range [IQR] for normally and non-normally distributed continuous variables and compared with appropriate parametric (including t-test and ANOVA) and non-parametric tests (including Mann–Whitney U and Kruskal–Wallis), respectively. Frequencies and percentages were reported for categorical variables, and were compared using chi-square test.

Incidence rates and 95% Confidence Intervals (CI) were calculated according to the number of events and person-years of follow-up,^26^ and multivariable Cox-regression analyses were performed to evaluate the effect of adherence to the ABC pathway on the risk of major outcomes, after adjustment for age (modelled as a linear variable), sex, type of AF, and major comorbidities (hypertension, diabetes mellitus, CHF, CAD, PAD and history of stroke/TIA); results were reported as Hazard Ratio (HR) and 95% CI. For the primary outcome, we also produced Kaplan–Meier curves to represent the cumulative hazard of patients, and survival distributions were compared using Log–Rank test. We also performed a quantile regression to estimate the delay of event (DoE)^27^, ^28^, ^29^ achieved with ABC adherence at 1 year of follow-up; for each comparison, we chose the quantile corresponding to the survival probability at 1-year of follow-up for the reference group.

We also performed three secondary analyses. In the first, we evaluated the effect of being adherent to 2–3 vs. 0–1 ABC criteria; in the second, we explored the effect of full adherence to ABC pathway; finally, we also assessed the contribution of different patterns of adherence to ABC criteria.

In the exploratory analyses, we also evaluated the impact of the ‘expanded’ ABC pathway on the risk of major outcomes, using multivariable Cox-regression models additionally adjusted also for the other comorbidities included in the ‘C’ criterion assessment. We also performed two sensitivity analysis: in the first, we included only those patients with at least 1 comorbidity at baseline (among hypertension, diabetes, CHF, CAD, PAD and history of stroke). In the second, we included only those patients enrolled in the Phase III of the GLORIA-AF Registry.

A two-sided p < 0.05 was considered statistically significant. All the analyses were performed using R 4.0.3 (R Core Team 2020, Vienna, Austria).

### Role of the funding source

This study was funded by Boehringer Ingelheim. The authors are solely responsible for the design and conduct of this study, all study analyses, the drafting and editing of the manuscript, and its final contents. G.F.R. and G.Y.H.L. had access to the dataset and decided to submit the manuscript; all authors supported the decision to submit for publication.

## Results

From the 36,617 patients originally enrolled in the GLORIA-AF Phase II and III Registry, 24,608 (67.2%; mean age 70.2 (10.3) years, 10,938 (44.4%) females) with complete data to evaluate ABC adherence and follow-up data for the primary outcome were included in this analysis. No significant differences in terms of age and CHA_2_DS_2_-VASc score were observed between included and excluded patients, while females were slightly more represented among those excluded, and other differences were observed regarding medical history and treatment received at baseline (Supplementary Table S2).

Among patients included, 707 (2.9%), 5285 (21.5%), 12,112 (49.2%), and 6504 (26.4%) were adherent to 0, 1, 2, and 3 ‘standard’ ABC criteria, respectively. Baseline characteristics according to the number of ABC criteria adherent are reported in Supplementary Table S3.

Adherence to an increasing number of criteria was associated with older age, higher prevalence of persistent or permanent AF as well as a clinical history less burdened with hypertension, CHF, CAD, diabetes, PAD, history of bleeding and thromboembolic events, chronic kidney disease and peptic disease, and lower CHA_2_DS_2_-VASc and HAS-BLED scores; on the other side, history of cancer increased with the number of criteria attained. The use of OAC (specifically, NOAC) increased with a higher number of ABC criteria fulfilled.

### Adverse outcomes according to ABC pathway adherence

**Fig. 1:**
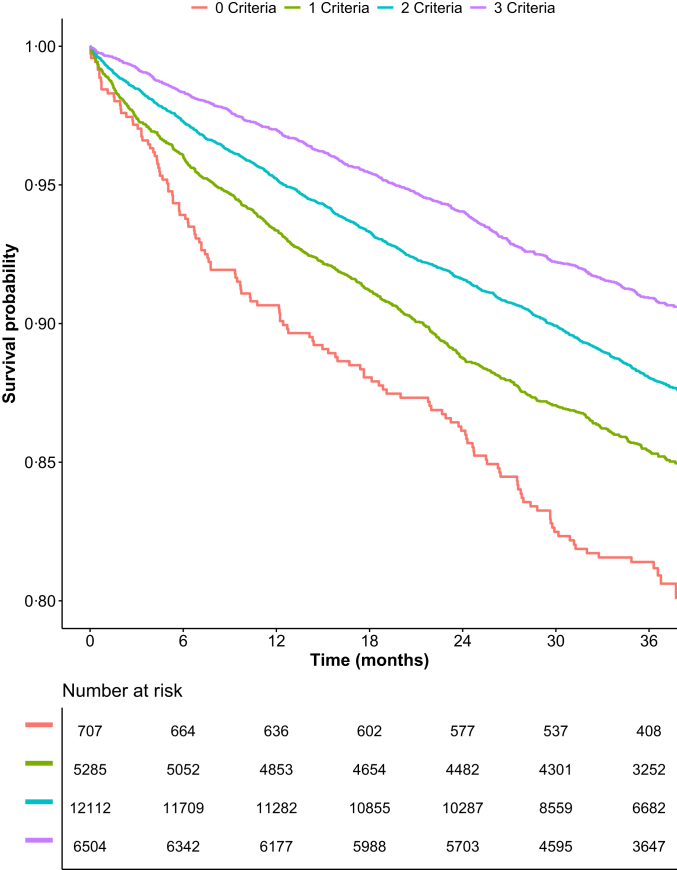
**Kaplan–Meier curves for the primary composite outcome of all-cause death and MACE according to the number of ABC criteria fulfilled**. p < 0.001 (Log–Rank test).

Over a median follow-up of 36.3 [IQR: 27.9–37.7] months, a total of 2822 (11.5%) events of the primary composite outcome of all-cause death or MACE occurred. Details on the number and incidence rates of the outcomes investigated are reported in Supplementary Table S4. Kaplan–Meier curves analysis for the primary composite outcome showed a progressively increasing rates of event for lower numbers of ABC pathway criteria attained (Fig. 1). The DoE analysis showed that adherence to an increasing number of criteria was associated with a significant gain of event-free survival at 1 year of follow-up (1 vs. 0 criteria: 248 [95% CI: 78–463] days, p = 0.012; 2 vs. 0 criteria: 487 [299–631] days, p < 0.0001; 3 vs. 0 criteria: 792 [452–1445] days, p = 0.0018; Fig. S1 in Supplementary Materials).

Adherence to an increasing number of ABC criteria was associated with a reduced risk of the primary composite outcome of all-cause death or MACE, with the highest reduction observed for those with full adherence (aHR: 0.54, 95% CI: 0.44–0.67, p < 0.0001) [Table 1; complete regression model for the primary outcome reported in Supplementary Table S5]. Similar results were observed for the risks of all-cause and cardiovascular death, MACE, and the net clinical outcome of all-cause death, MACE and major bleeding. The risk of major bleeding was not statistically different between any of the groups investigated, when compared to patients adherent to 0 criteria.

**Table 1:** Risk of major outcomes according to the adherence to the ABC pathway (n = 24,608).

Outcome	Number of criteria				0-1 vs. 2–3 criteria		Full vs. incomplete adherence	
	0 criteria (n = 707) (Ref.)	1 criteria (n = 5285) aHR [95% CI]^a^	2 criteria (n = 12,112) aHR [95% CI]^a^	3 criteria (n = 6504) aHR [95% CI]^a^	0-1 criteria (n = 5992) (Ref.)	2-3 criteria (n = 18,616) aHR [95%CI]^a^	Incomplete adherence (n = 18,104) (Ref.)	Full adherence (n = 6504) aHR [95%CI]^a^
Primary outcome								
All-cause death and MACE	Ref.	**0.69 [0.57–0.84]**	**0.58 [0.48–0.70]**	**0.54 [0.44–0.67]**	Ref.	**0.79 [0.72–0.86]**	Ref.	**0.87 [0.79–0.97]**
Secondary outcomes								
All-cause death	Ref.	**0.73 [0.58–0.92]**	**0.61 [0.49–0.76]**	**0.58 [0.46–0.74]**	Ref.	**0.79 [0.72–0.87]**	Ref.	**0.89 [0.79–1.00]**
CV death	Ref.	**0.62 [0.45–0.87]**	**0.46 [0.33–0.65]**	**0.44 [0.30–0.64]**	Ref.	**0.69 [0.59–0.82]**	Ref.	0.84 [0.68–1.04]
Stroke	Ref.	0.71 [0.47–1.08]	**0.63 [0.42–0.95]**	**0.51 [0.33–0.79]**	Ref.	**0.81 [0.67–0.97]**	Ref.	**0.77 [0.62–0.95]**
Thromboembolism	Ref.	0.74 [0.51–1.07]	**0.65 [0.46–0.93]**	**0.54 [0.37–0.79]**	Ref.	**0.81 [0.69–0.95]**	Ref.	**0.78 [0.65–0.94]**
MACE	Ref.	**0.64 [0.50–0.82]**	**0.52 [0.40–0.66]**	**0.46 [0.35–0.60]**	Ref.	**0.74 [0.66–0.84]**	Ref.	**0.82 [0.71–0.95]**
Myocardial infarction	Ref.	0.69 [0.43–1.11]	**0.59[0.37–0.93]**	0.64 [0.38–1.06]	Ref.	0.82 [0.65–1.03]	Ref.	1.01 [0.77–1.33]
Major bleeding	Ref.	1.01 [0.63–1.61]	1.10 [0.70–1.74]	1.11 [0.69–1.78]	Ref.	1.10 [0.92–1.31]	Ref.	1.04 [0.87–1.24]
All-cause death, stroke, and major bleeding	Ref.	**0.78 [0.64–0.96]**	**0.69 [0.57–0.85]**	**0.67 [0.54–0.82]**	Ref.	**0.86 [0.79–0.93]**	Ref.	0.92 [0.83–1.01]
All-cause death, MACE, and major bleeding	Ref.	**0.75 [0.62–0.91]**	**0.67 [0.56–0.80]**	**0.64 [0.53–0.78]**	Ref.	**0.85 [0.78–0.92]**	Ref.	0.92 [0.83–1.01]

Bold text depicts statistically significant results at p < 0.05 level.

aHR = adjusted hazard ratio; CI = confidence interval; CV = cardiovascular; MACE = major adverse cardiovascular events; Ref. = Reference group.

a Adjusted for age, sex, type of atrial fibrillation, hypertension, diabetes mellitus, chronic heart failure, coronary artery disease, peripheral artery disease, history of stroke/transient ischemic attack.

Both adherence to 2 or 3 ABC criteria (compared to adherence to 0–1 criteria), and full adherence (compared to incomplete adherence) were associated with a reduction of the risk for the primary outcome (Table 1), and with a significant DoE at 1 year of follow-up for the primary outcome (285 [231–343] days, p < 0.0001 and 298 [228–369] days, p < 0.0001; Figs. S2 and S3 in Supplementary Materials, respectively).

Compared to incomplete adherence, full adherence to ABC pathway was associated with a reduction in the risk of all-cause death, stroke, thromboembolism, and MACE, while no statistically significant differences were observed for the other outcomes investigated. Patients adherent to at least 2 ABC criteria showed a lower risk of all the secondary outcomes, except for major bleeding and myocardial infarction.

**Fig. 2:**
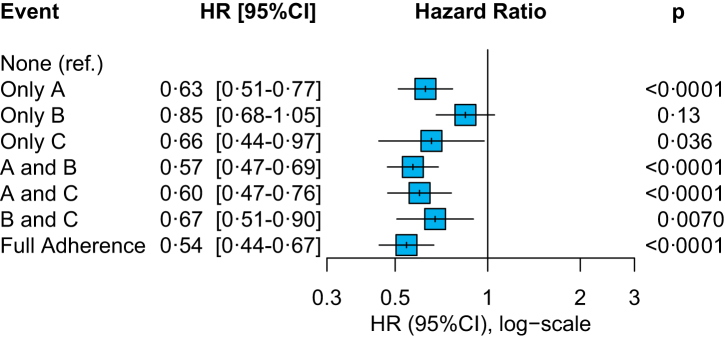
**Pattern of adherence to the ABC pathway and risk of the primary outcome of all-cause death and MACE**. Adjusted for age, sex, type of atrial fibrillation, hypertension, diabetes mellitus, chronic heart failure, coronary artery disease, peripheral artery disease, history of stroke/transient ischemic attack. Ref. = Reference group.

The analysis on the pattern of ABC adherence and the risk of the primary outcome is shown in Fig. 2. Among those patients adherent to only 1 criterion, attainment of ‘A’ or ‘C’ criteria was associated with a significant reduction of the composite outcome of all-cause death and MACE (aHR: 0.63, 95% CI: 0.51–0.77 and aHR: 0.66, 95% CI: 0.44–0.97 respectively), while only a non-significant trend was observed for adherence to ‘B’ criterion only. Adherence to each combination of at least 2 criteria was also associated with a significantly lower risk, with greater benefit observed for ‘A-B’ and ‘A-C’ criteria combinations (aHR: 0.57, 95% CI: 0.47–0.69, and aHR: 0.60, 95% CI: 0.47–0.76, respectively). Consistent with the primary analysis, complete ABC pathway adherence was associated with the greatest risk reduction.

### Expanded ABC pathway

Among patients included, 761 (3.1%), 5580 (22.7%), 12,373 (50.3%), and 5894 (24.0%) were adherent to 0, 1, 2, and 3 ‘expanded’ ABC criteria, respectively.

The analysis on the impact of the ‘expanded’ version of the ABC pathway showed similar results compared to the results observed for the ‘standard’ ABC pathway (Supplementary Table S6), although with trends toward a lower magnitude of effect. The risk of the primary outcome and most secondary outcomes were progressively reduced with the attainment of an increased number of ABC criteria, and adherence to at least 2 ‘expanded’ ABC criteria was associated with a consistent reduction of all the outcomes investigated, except for major bleeding (Supplementary Table S6). A trend towards reduction of the risk of primary outcome was found among those with full adherence to ‘expanded’ ABC pathway (compared to incomplete adherence), while the risk of thromboembolism was significantly reduced (aHR: 0.82, 95% CI: 0.68–0.99, p = 0.038).

### Sensitivity analyses

We evaluated the impact of the ‘standard’ ABC pathway among the 21,579 (87.7%) patients with at least 1 comorbidity among hypertension, diabetes, CAD, CHF, PAD and history of stroke/TIA. The results were consistent with the primary analyses, with a significant reduction for the risk of primary outcome which was greater as the number of ABC adherent criteria increased (Table 2).

**Table 2:** Risk of major outcomes according to the adherence to the ABC pathway in those with at least 1 comorbidity at baseline (n = 21,579).

Outcome	Number of criteria				0-1 vs. 2–3 criteria		Full vs. incomplete adherence	
	0 criteria (n = 707) (Ref.)	1 criteria (n = 5037) aHR [95% CI]^a^	2 criteria (n = 11,026) aHR [95% CI]^a^	3 criteria (n = 4809) aHR [95% CI]^a^	0-1 criteria (n = 5744) (Ref.)	2-3 criteria (n = 15,835) aHR [95% CI]^a^	Incomplete adherence (n = 16,770) (Ref.)	Full adherence (n = 4809) aHR [95%CI]^a^
Primary outcome								
All-cause death and MACE	Ref.	**0.70 [0.58–0.85]**	**0.59 [0.49–0.71]**	**0.53 [0.43–0.65]**	Ref.	**0.78 [0.72–0.86]**	Ref.	**0.84 [0.75–0.94]**
Secondary outcomes								
All-cause death	Ref.	**0.74 [0.59–0.92]**	**0.61 [0.49–0.77]**	**0.57 [0.45–0.73]**	Ref.	**0.79 [0.72–0.88]**	Ref.	**0.87 [0.76–0.99]**
CV death	Ref.	**0.63 [0.45–0.89]**	**0.47 [0.34–0.65]**	**0.44 [0.30–0.65]**	Ref.	**0.69 [0.58–0.81]**	Ref.	0.83 [0.66–1.04]
Stroke	Ref.	0.72 [0.47–1.09]	**0.64 [0.42–0.96]**	**0.49 [0.32–0.77]**	Ref.	**0.80 [0.66–0.97]**	Ref.	**0.74 [0.58–0.93]**
Thromboembolism	Ref.	0.75 [0.52–1.08]	**0.66 [0.46–0.94]**	**0.53 [0.36–0.79]**	Ref.	**0.81 [0.69–0.95]**	Ref.	**0.77 [0.63–0.94]**
MACE	Ref.	**0.65 [0.50–0.83]**	**0.52 [0.41–0.66]**	**0.44 [0.33–0.58]**	Ref.	**0.73 [0.65–0.82]**	Ref.	**0.78 [0.66–0.91]**
Myocardial infarction	Ref.	0.71 [0.44–1.14]	**0.58 [0.36–0.91]**	0.62 [0.37–1.03]	Ref.	**0.78 [0.62–0.99]**	Ref.	0.98 [0.72–1.32]
Major bleeding	Ref.	1.00 [0.63–1.60]	1.11 [0.71–1.75]	1.13 [0.70–1.81]	Ref.	1.11 [0.93–1.33]	Ref.	1.05 [0.86–1.27]
All-cause death, stroke and major bleeding	Ref.	**0.78 [0.64–0.96]**	**0.70 [0.57–0.85]**	**0.67 [0.54–0.82]**	Ref.	**0.86 [0.79–0.94]**	Ref.	0.91 [0.82–1.01]
All-cause death, MACE and major bleeding	Ref.	**0.76 [0.63–0.91]**	**0.67 [0.56–0.81]**	**0.64[0.52–0.78]**	Ref.	**0.85 [0.78–0.92]**	Ref.	**0.90 [0.81–0.99]**

Bold text depicts statistically significant results at p < 0.05 level.

aHR = adjusted hazard ratio; CI = confidence interval; CV = cardiovascular; MACE = major adverse cardiovascular events; Ref. = Reference group.

a Adjusted for age, sex, type of atrial fibrillation, hypertension, diabetes mellitus, chronic heart failure, coronary artery disease, peripheral artery disease, history of stroke/transient ischemic attack.

**Fig. 3:**
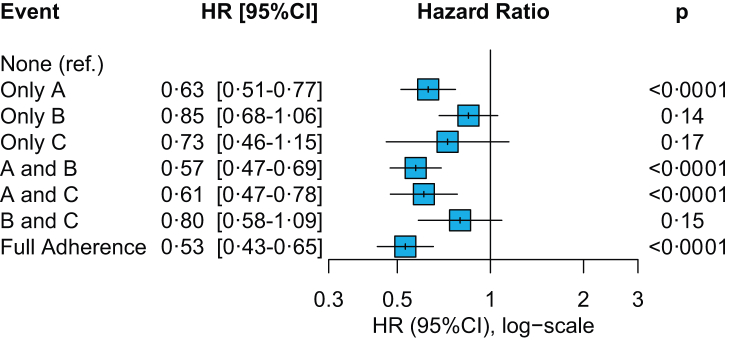
**Pattern of adherence to the ABC pathway and risk of the primary outcome of all-cause death and MACE in patients with at least 1 comorbidity at baseline**. Adjusted for age, sex, type of atrial fibrillation, hypertension, diabetes mellitus, chronic heart failure, coronary artery disease, peripheral artery disease, history of stroke/transient ischemic attack. Ref. = Reference group.

Adherence to at least 2 ABC criteria, and full ABC pathway adherence were both associated with significant reductions of the risk of the primary outcome (aHR: 0.78, 95% CI: 0.72–0.86 and aHR: 0.84, 95% CI: 0.75–0.94, respectively). The analysis on the pattern of ABC adherence (Fig. 3) consistently showed a significant reduction in the risk of the primary outcome among those patients adherent to ‘A’ criterion, and the combinations of ‘A-B’ and ‘A-C’ criteria. Complete adherence showed the greatest magnitude of risk reduction (aHR: 0.53, 95% CI: 0.43–0.65, p < 0.0001).

We finally performed a sensitivity analysis including only those patients enrolled in the Phase III of the GLORIA-AF Registry (n = 21,147, 85.9%). The results of this analysis are reported in Supplementary Table S7 in Supplementary Materials, and showed broadly consistent results compared to the primary analysis.

## Discussion

In this analysis, which currently represents the largest cohort-study assessment of the effect of the ABC pathway in patients with AF, our principal findings are as follows: a) adherence to the ABC pathway was associated with a reduced risk of the composite outcome of death and MACE, with the magnitude of benefit that increased according to the number of ABC criteria attained; b) adherence to the ABC pathway was associated with a lower risk of cardiovascular death, stroke, thromboembolism, and the net composite outcome of death, MACE and major bleeding; c) while full adherence to ABC pathway showed the largest risk reduction, a significant risk reduction was shown for most of the outcomes considered when at least 2 ABC criteria were attained, with the ‘A’ and ‘C’ criteria being the most important drivers of ABC pathway effect.

In our DoE analysis, we also observed that patients who were adherent to the ABC pathway experienced a significant increase in event-free survival, which was progressively higher as the number of criteria attained increased. Our additional analyses also showed that the ‘expansion’ to the non-cardiovascular comorbidities included in the ‘C’ criterion assessment provided broadly similar results, underlining the importance of comorbidities optimization beyond the most frequently found cardiovascular conditions seen in patients with AF. Finally, our sensitivity analyses showed that the effect of ABC pathway was maintained even in those with at least one concomitant disease, reinforcing the importance of implementing an integrated care approach in these patients.

Over the last decade, the introduction of the ‘integrated care’ concept has been one of the most relevant innovations in AF^9^ and other chronic long-term conditions,^30^^,^^31^ acknowledging the need for a new, comprehensive model of care for such patients, who are becoming increasingly complex and with unmet health needs.^32^

Since its first proposal, the efficacy of the ABC pathway has been consistently shown in several retrospective analyses,^10^, ^11^, ^12^^,^^33^ and also in a prospective cluster randomised trial.^13^ However, granular data on the impact of adherence to a different number of ABC criteria, as well as on pattern of ABC adherence, are scarce and limited by the sample sizes of the studies which were often conducted in specific countries or regions. Also, while the importance of cardiovascular comorbidity optimization was consistently shown in previous research, the contribution of the optimal management of non-cardiovascular comorbidities was unclear.

Our manuscript fills these evidence gaps and provides essential insights on the effectiveness of an ABC pathway-adherent management in a contemporary global prospective cohort of newly diagnosed patients with AF. Our findings, while broadly confirming previous results, also shows that the ABC pathway had an ‘exposure-effect’ impact, with the magnitude of risk reduction increasing with the number of ABC criteria met. Moreover, a significant delay of the primary composite outcome after the first year of follow-up was observed for those patients adherent to at least 1 ABC criteria, with a delay of more than two years observed among those adherent to all the 3 ABC criteria.

Furthermore, the pattern of ABC adherence demonstrated that the ‘A’ and ‘C’ criteria were the most strongly associated with risk reduction. Also, it is important to underline how the event-free survival was similarly increased in patients with 2–3 ABC criteria attained and in those fully adherent (compared to all of those not fully adherent). Taken together, these findings underline how also an incomplete adherence to an integrated care approach (which may be related to challenges in full implementation) impacts on clinical outcomes in patients with AF, and that the holistic management aiming at comorbidities control represents an effective strategy to reduce the risk of major outcomes among these patients.

Apart from a consistent reduction for most of the outcomes investigated (including stroke, thromboembolism, and MACE) also beyond what has been previously demonstrated in a recent systematic review and meta-analysis,^14^ we did not find any significant difference in the risk of major bleeding, across all levels of adherence to ABC pathway. This observation can be partly explained by the low incidence of major bleeding observed across all levels of ABC pathway adherence. This may reflect the higher adoption of NOACs in clinical practice,^34^ and the relatively low risk of the cohort investigated.

This analysis is also the first to expand the pool of non-cardiovascular comorbidities evaluated for optimal management in the ‘C’ criterion. The inclusion of the optimal management of hyperthyroidism, hyperlipidaemia, and dyspeptic diseases (which are all common in patients with AF) among the qualifying items for the ‘expanded’ version of the ABC pathway led to broadly similar results and estimates compared to the ‘standard’ version of the ABC pathway, although with trends towards reduced magnitude of the effect, which may be due to the lower number of patients who fulfilled all the 3 criteria. This reinforces the concept that an holistic management of patients with AF that include all the concurrent diseases, and not only the cardiovascular ones, is able to improve outcomes. As multimorbidity is increasingly found among subjects with AF,^23^ this represents one key message for clinical practice and future research; further studies are needed to confirm and expand these observations.

Our sensitivity analysis also showed a consistent effect of the ABC pathway among patients with at least one concurrent disease. As this cohort included newly diagnosed patients with AF, taken together these results underline how the implementation of a comprehensive management may be particularly useful among those who present with concurrent comorbidities at baseline, and are therefore more prone to the risk of adverse outcomes. Also, these results strengthen the impact of ABC pathway, and confirm the previous data about its effectiveness in patients with multimorbidity.^11^^,^^35^

Our study has several strengths. This analysis represents the largest assessment of the ABC pathway in a cohort study thus far, and the first to analyse an ‘expanded’ version of the ABC pathway enriched with additional non-cardiovascular comorbidities. Furthermore, this analysis included patients with newly diagnosed AF, enrolled globally. Compared to previous analyses, we refined the definition of “optimal management” used for some of the comorbidities assessed, including the use of baseline blood pressure control, symptomatic status, and presence of end-organ damage for the definition of optimal control of hypertension, CHF and diabetes, respectively. This has allowed the development of definitions that were more representative of the ‘actual control’, rather than the intention of control. However, this approach was not suitable for all the comorbidities investigated, thus representing a limitation common to all the retrospective analyses on the efficacy of ABC pathway.

Nevertheless, our study has also several limitations. Given its observational, post-hoc nature of a prospectively collected dataset, we may have limited power to detect differences in groups not specified in the original study protocol. Moreover, we included only patients with complete data on the adherence to the ABC pathway and follow-up data on the primary outcome, and this may have introduced some degree of selection bias in our analysis; indeed, some differences were observed among those included vs. excluded in this analysis, although these were expected also considering the differences in the study design of Phase II and Phase III of the GLORIA-AF Registry. We evaluated the adherence to ABC pathway, and its impact on major outcomes retrospectively; furthermore, given the data available, the adherence to the ‘C’ criterion was based mainly on the ‘attempt’ to optimal management (i.e., treatment prescribed at baseline), as we did not have data on the attainment of treatment targets. Although this is a limitation common to several other analyses conducted on the ABC pathway, this may have introduced bias in the evaluation of our results. Imbalance in the distribution of baseline comorbidities may have biased our results; however, we made our best efforts to control our analyses for the contribution of potential moderators using covariated-adjusted models, which are recognised among the most suitable techniques to adjust for confounding in observational studies.^36^ However, we cannot exclude the contribution of unaccounted confounders in the results observed. Moreover, in the ‘expanded’ version of the ABC pathway, we evaluated optimal management of three additional comorbidities, that were chosen also according to availability of data to capture their optimal management. Other non-cardiovascular comorbidities may have a stronger impact on the prognosis of patients with AF, and further studies are required to evaluate the impact of their optimal management. We also cannot exclude potential regional-based differences in the efficacy of the ABC pathway; however, our study was not sufficiently powered to explore these differences reliably (especially for some specific regions), and further studies are required to clarify this issue. Finally, our results on secondary outcomes were not adjusted for multiple comparisons, and therefore should be interpreted with caution.

In conclusion, in this large prospective global registry, adherence to ABC pathway in patients with AF was associated with a reduced risk of major adverse events, including mortality, thromboembolism, and MACE. The greatest magnitude of benefit was observed among those managed as fully adherent to ABC pathway. The expansion of non-cardiovascular comorbidities assessed in the ‘C’ criterion, and the analysis among those with at least one concurrent disease provided consistent results.

## Contributors

G.F.R., M.P. and G.Y.H.L. conceived and design the analysis, and verified the underlying data; G.F.R. analysed data; G.F.R. and M.P. interpreted data and drafted the manuscript; N.B., W.Y.D., G.B., M.V.H. and G.Y.H.L. revised manuscript and gave relevant intellectual contribution. G.F.R. and G.Y.H.L. had access to the dataset, all authors read and approved the final manuscript, and accept responsibility to submit for publication.

## Data sharing statement

Data supporting this study by the data contributors Boehringer Ingelheim, and were made and are available through Vivli, Inc.

## Declaration of interests

G.Y.H.L. has been consultant and speaker for BMS/Pfizer, Boehringer Ingelheim and Daiichi-Sankyo. No fees are directly received personally. All the disclosures happened outside the submitted work. G.B. received small speaker's fees from Medtronic, Boston, Boehringer Ingelheim and Bayer. M.V.H. has been receiving research grants from the Dutch Healthcare Fund, Dutch Heart Foundation, BMS-Pfizer, Bayer Healthcare and Boehringer Ingelheim and consulting fees from BMS-Pfizer, Bayer Healthcare and Boehringer Ingelheim. All other authors have nothing to declare.
